# Advancements and Prospects of pH-Responsive Hydrogels in Biomedicine

**DOI:** 10.3390/gels11040293

**Published:** 2025-04-15

**Authors:** Ke Gao, Ke Xu

**Affiliations:** 1 Zhejiang Provincial Engineering Research Center of New Technologies and Applications for Targeted Therapy of Major Diseases, College of Life Science and Medicine, Zhejiang Sci-Tech University, Hangzhou 310018, China; 2022332864026@mails.zstu.edu.cn; 2 Key Laboratory of Advanced Textile Materials & Manufacturing Technology, Ministry of Education, Zhejiang Sci-Tech University, Hangzhou 310018, China

**Keywords:** pH-responsive, hydrogels, microenvironments, fabrication process, application

## Abstract

As an intelligent polymer material, pH-sensitive hydrogels exhibit the capability to dynamically sense alterations in ambient pH levels and subsequently initiate corresponding physical or chemical responses, including swelling, contraction, degradation, or ion exchange. Given the significant pH variations inherent in human pathophysiological microenvironments, particularly in tumor tissues, inflammatory lesions, and the gastrointestinal system, these smart materials demonstrate remarkable application potential across diverse domains such as targeted drug delivery systems, regenerative medicine engineering, biosensing, and disease diagnostics. Recent breakthroughs in nanotechnology and precision medicine have substantially propelled advancements in the design and application of pH-responsive hydrogels. This review systematically elaborates on the current research progress and future challenges regarding pH-responsive hydrogels in biomedical applications, with particular emphasis on their stimulus–response mechanisms, fabrication methodologies, multifunctional integration strategies, and application scenarios.

## 1. Introduction

Hydrogels are three-dimensional polymeric network systems formed through chemical bonds (covalent bonds) and physical interactions (hydrogen bonds, Coulombic forces, coordination bonds, etc.) [1]. Typically utilizing water as the liquid medium without dissolving in aqueous solutions [2], these materials maintain structural integrity while enabling energy, mass, and information exchange with external environments [3,4]. Characterized by a high water absorption capacity, moisture retention, and excellent biocompatibility, hydrogels demonstrate significant potential for biomedical applications due to their capacity for drug encapsulation [5,6]. Current medical applications encompass drug delivery carriers, corneal contact lenses, scaffolds for bone and soft tissue regeneration, as well as wound healing and anti-infection dressings [3,7,8,9]. However, conventional hydrogels exhibit limited environmental responsiveness, particularly in adapting to dynamic pathophysiological conditions at specific tissue sites, thereby constraining their clinical utility [10].

Smart responsive hydrogels represent a novel class of gel materials capable of exhibiting adaptive behaviors in response to environmental variations [11]. These materials undergo conformational physical or chemical changes upon external stimuli, thereby manifesting characteristic responses such as swelling, contraction, or degradation [12]. Typically, these gels are primarily prepared using two methods: physical crosslinking and chemical crosslinking. Physical crosslinking mainly relies on interactions such as hydrogen bonding, ionic chelation, hydrophobic association, and chain entanglement. Chemical crosslinking of hydrogels involves processes like polymerization reactions, Michael addition, click chemistry using Schiff bases, and enzyme-mediated reactions. These synthetic methods have been thoroughly discussed in earlier reviews [13,14,15,16,17].

Generally, pathological biochemical processes in human physiology are frequently accompanied by localized pH alterations at disease sites [18,19]. Consequently, the development of pH-responsive hydrogels and their biomedical applications for therapeutic interventions or physiological monitoring in various tissues and organs has attracted significant research attention [20,21]. However, lesion sites in the human body are frequently subjected to complex internal environmental factors (e.g., low pH, elevated ROS levels, high glucose concentrations, and overexpressed enzymes) or external conditions (e.g., temperature, light, and magnetic fields), which contribute to persistent therapeutic challenges [22,23,24,25]. Consequently, conventional pH-responsive hydrogels no longer suffice for current clinical demands. To address this, an increasing number of pH-sensitive dual- or multi-responsive hydrogels have been developed in recent years, offering novel strategies for future clinical applications. In light of these advancements, this review analyzes and synthesizes literature from the past five years focusing on pH-responsive hydrogels, aiming to elucidate their evolving roles in biomedical research and therapy.

**Figure 1:**
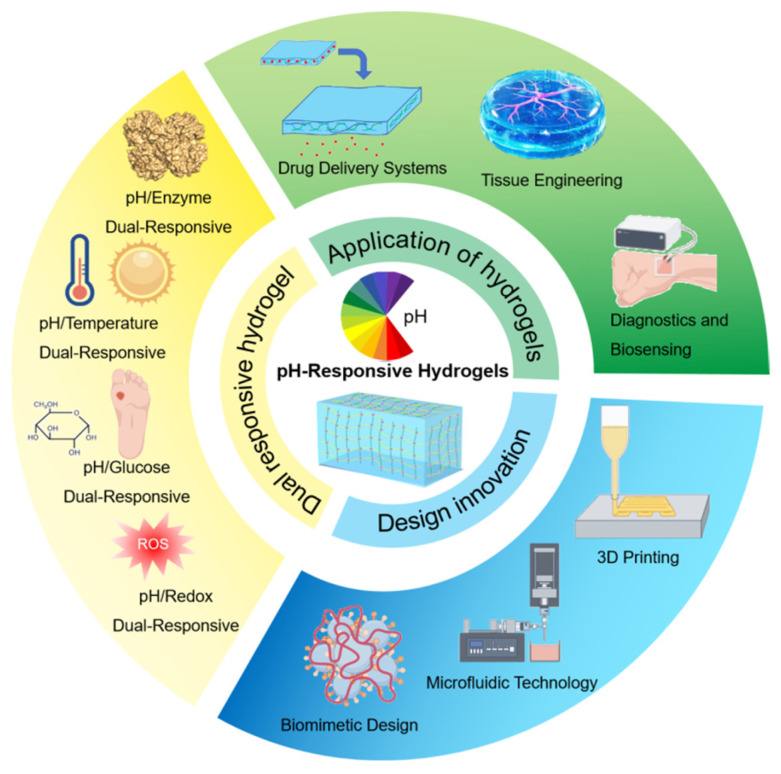
Preparation, design, and applications of pH-responsive hydrogels.

Previous literature reviews have predominantly focused on the fabrication principles and drug delivery applications of pH-responsive hydrogels [26,27,28,29]. Building upon this foundation, the present study employs bibliometric analysis to not only systematically elaborate on design strategies for pH-sensitive multi-responsive hydrogels but also extend the discussion to their multifunctional integration, advanced fabrication techniques, and bioinspired design innovations(as shown in Figure 1). In summary, the review aims to present a holistic perspective on the current research landscape, recent advancements, persistent challenges, and future prospects within this rapidly evolving field.

## 2. Bibliometric Analysis

Bibliometrics is a significant branch of informatics, representing a discipline that employs mathematical and statistical methods to conduct quantitative and visual analyses of the characteristics of literature and their developmental patterns. Its core objective is to uncover the distribution, relationships, and evolutionary trends of scholarly outputs. Additionally, it facilitates the derivation, induction, and mining of new knowledge from vast repositories of existing knowledge [30,31].

In recent years, significant advancements have been achieved in international research on pH-responsive hydrogel materials, with a notable increase in publications focusing on their biomedical applications. Consequently, this study primarily adopts bibliometric approaches to analyze and summarize current research hotspots, developmental trajectories, and application prospects of pH-responsive medical hydrogels. Methodologically, specialized bibliometric software is utilized to conduct statistical analyses regarding publication outputs associated with pH-responsive hydrogels, encompassing dimensions such as author contributions, national/regional distributions, institutional affiliations, and journal sources.

### 2.1. Software Overview

The bibliometric software packages employed in this study included VOSviewer (1.6.20.0) and Bibliometrix (4.1.2). These tools, developed based on Java and R programming languages, respectively, constitute comprehensive bibliometric analysis toolkits that integrate data collection, analysis, and visualization functionalities. Both platforms enable researchers to conduct in-depth investigations of the academic literature, knowledge networks, collaborative relationships, and research hotspots [32]. They demonstrate compatibility with mainstream academic databases, such as Web of Science and Scopus, supporting data importation through multiple reference manager file formats. Characterized by intuitive interfaces and powerful functionalities, these software packages have become indispensable visualization tools in academic research [33,34].

### 2.2. Data Screening

This study primarily selected the Web of Science (WOS) Core Collection as the principal data source and set the SCI-EXPANDED and SSCI databases as the indexing scope. The search strategy was defined as TS = (pH responsive) AND (hydrogel).The temporal scope spanned from 1 January 2019 to 31 December 2024. The initial search yielded 2465 journal articles. Subsequent data cleansing procedures were implemented to eliminate entries irrelevant to the research theme, resulting in 1948 valid articles ultimately obtained.

**Figure 2:**
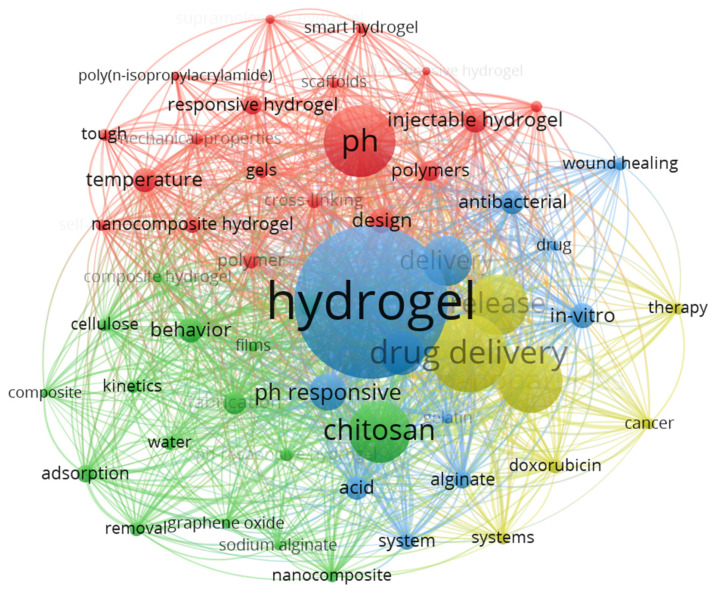
Relationships among literature keywords generated using VOSViewer software.

Meanwhile, VOSViewer software was utilized to conduct a co-occurrence analysis of keywords from the previously mentioned 1948 articles to better understand how various keywords are interconnected. The type of analysis selected was Co-occurrence; the unit of analysis was All keywords; and the counting method was Full counting. Among the 6414 keywords, the top 54 with the highest frequency (with a minimum occurrence rate set at 40 times) were selected. Figure 2 displays the network diagram of all keywords, divided into 4 groups, with different colors representing different nodes. Lines indicate connections, and the thickness of the lines between nodes represents the strength of their relationship. Nodes represent keywords, and the size of the nodes is proportional to their frequency in the literature [35]. Through this visual analysis, it becomes easier to intuitively grasp the relationships between these keywords and their significance within the network. The figure reveals that the four keywords (including hydrogel, pH, drug delivery, and chitosan) have the highest frequency of occurrence in this field. This indicates that research centered on pH-responsive hydrogels typically uses chitosan as a carrier material to explore advancements in the drug delivery domain. Additionally, it is observed that keywords such as sodium alginate, cellulose, injectable gels, cancer, wound healing, and doxorubicin also have high frequencies, following closely behind. This reflects significant information about pH-responsive hydrogels in terms of commonly used materials, functionalities, and applications, as well as the degree of correlation between these keywords.

### 2.3. Statistical Analysis

During the literature statistical phase, the screened 1948 pieces of literature were exported in three batches using the Plaintext file format, with exported records encompassing complete metadata and references. Bibliometric analysis was conducted using the Bibliometrix software to perform a comprehensive quantitative assessment. This process yielded a general summary of key metrics, including fundamental indicators such as annual publication volume, country-specific contributions, average citations per paper, annual growth rate (%), and journal-specific distributions.

**Figure 3:**
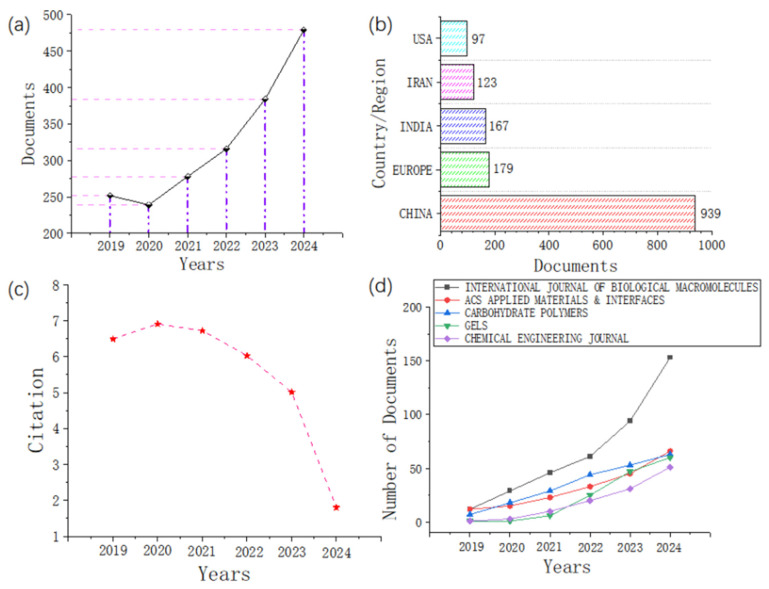
Bibliometric analysis of literature based on the WOS database, including annual publication output (**a**), top five countries in publication output (**b**), average annual citation per document (**c**), and growth trend of the top five journals in publication output (**d**).

As illustrated in Figure 3a, the annual publication count demonstrated a consistent upward trajectory from 2019 (252 articles) to 2024 (479 articles), indicative of growing academic interest in pH-responsive hydrogels for biomedical applications. Figure 3b presents the geographical distribution of publications, revealing China, Europe, India, Iran, and the United States as the top five contributing nations. Citation analysis (Figure 3c) demonstrated sustained high citation rates from 2019 (6.49 citations/year) to 2022 (6.03 citations/year), with subsequent declines in 2023 (5.01) and 2024 (1.80) attributable to the recency of publications during these periods. Figure 3d shows the top five journals with the highest number of publications in recent years. These journals are the *International Journal of Biological Macromolecules*, *ACS Applied Materials & Interfaces*, *Carbohydrate Polymers*, *Gels*, and *Chemical Engineering Journal*. The data indicate that the number of publications in these journals is increasing at a relatively fast pace.

## 3. Principles of pH-Responsive Hydrogels

**Figure 4:**
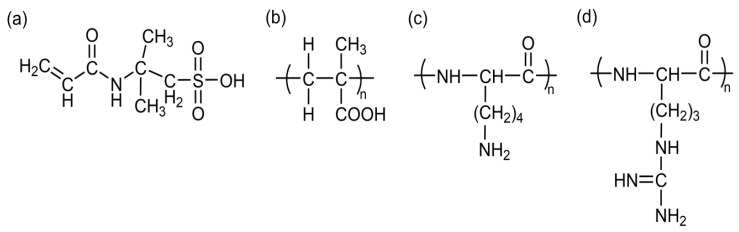
Chemical structures of pH-sensitive polymers: (**a**) 2-acrylmido-2-methylpropylsulfonic acid; (**b**) poly(methacrylic acid); (**c**) poly (L-lysine); (**d**) poly (arginine).

pH-responsive hydrogels were first discovered by Tanaka’s research group during their investigation of the swelling behavior of aged polyacrylamide gels [36]. This type of hydrogel exhibits variable swelling degrees in response to pH changes, subsequently being classified as pH-sensitive gels by researchers [37,38]. Structurally, these hydrogels typically contain hydrolytically labile or protonatable functional groups, including sulfonic acid, carboxylic acid, primary amine, and secondary amine groups (as illustrated in Figure 4) [39]. These functional groups undergo protonation/deprotonation reactions under different pH conditions, leading to significant alterations in hydrogel volume, morphology, and physical properties such as swelling/deswelling behaviors [25]. Macroscopically, these transformations enable the hydrogels to modulate their volumetric dimensions [40], degradation kinetics [41], and drug release profiles [42] in accordance with environmental pH variations. The pH-dependent responsiveness arises from the ionization equilibrium of functional groups, which modifies the osmotic pressure and electrostatic repulsion within the polymer network, ultimately governing the hydrogel’s macroscopic behavior.

**Figure 5:**
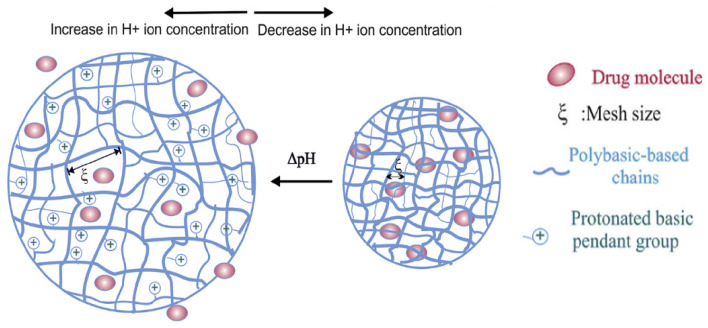
Swelling behavior of pH-responsive hydrogels under acidic conditions [46]. Copyright Bazban-Shotorbani S. et al. Journal of Controlled Release.

The pH-responsive dimensional changes are primarily reflected in the swelling behavior of hydrogels [43]. Specifically, the swelling ratio of hydrogels significantly varies with pH changes, leading to alterations in pore size within the material. This affects pH-responsive drug delivery or signal transmission. Different swelling behaviors are typically attributed to the protonation, deprotonation, and charge repulsion of functional groups within the material components [44,45]. Generally, these hydrogels can be classified into two major categories: polyacidic (or anionic) hydrogels and polybasic (or cationic) hydrogels. As shown in Figure 5, polybasic hydrogels relax at relatively high pH levels because their basic side groups are protonated and deionized. However, as the pH decreases, these groups start to ionize, which can cause the hydrogels to swell, thereby triggering drug release [46]. For example, the pH-sensitive composite hydrogelsprepared by Al-Arjan WS et al. [47] exhibit minimal swelling under acidic and alkaline conditions but demonstrate remarkable swelling at pH 7.4, leading to high curcumin release.The hydrophilic groups on the structure of bacterial cellulose (BC), polyvinyl alcohol (PVA), and graphene oxide (GO) cause swelling due to hydrogen-bonding interactions. In a neutral medium, water infiltrates the internal void spaces of the hydrogel. Additionally, functional groups such as carboxyl groups in BC and GO become protonated, generating greater electrostatic repulsion that leads to expansion.

**Figure 6:**
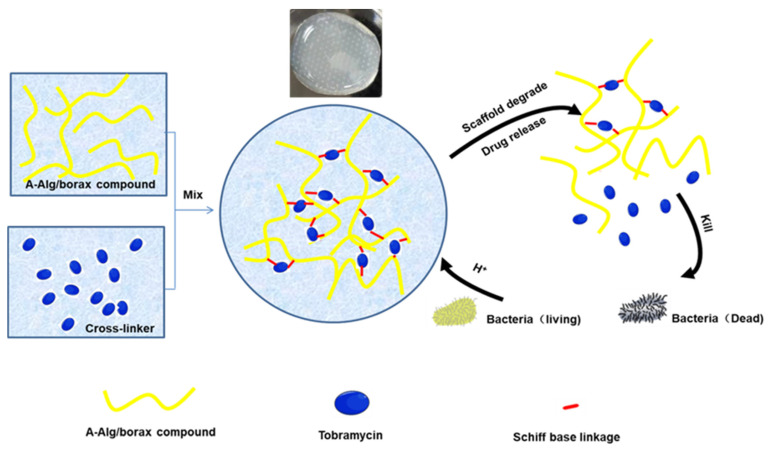
Degradation and drug release behavior of pH-responsive hydrogels under acidic conditions.

pH-responsive degradation is an effective strategy for controlling drug release [48]. This type of response typically involves pH-sensitive bonds such as imine, Schiff base, or ester bonds to achieve controlled degradation. Ideally, when bacterial metabolism creates an acidic microenvironment, it triggers the degradation of pH-responsive hydrogels, subsequently releasing drugs to eliminate bacteria [49]. Once the tissue’s pH returns to normal, the degradation process halts [50]. As the degradation rate increases, the strength of the hydrogel’s three-dimensional network weakens, leading to larger internal pores [51]. The experimental results from our previous study, as illustrated in Figure 6, demonstrate that tobramycin (represented by blue ellipses), an aminoglycoside antibiotic, can initiate a sol–gel transition through the formation of dynamic Schiff base bonds (denoted by red dots) between its amino groups and aldehyde moieties (indicated by yellow lines) in oxidized sodium alginate. Furthermore, bacterial metabolism in both solid and liquid culture media was observed to decompose organic compounds into lactic acid and carbonic acid, creating localized acidic microenvironments. The resultant H^+^ ions effectively cleave the Schiff base crosslinking points within the hydrogel network. This pH-responsive bond dissociation mechanism enables the controlled release of tobramycin for bactericidal action, concomitant with hydrogel matrix degradation [45]. In this process, tobramycin serves both as a crosslinking agent during gel formation and as a model drug during gel degradation.

## 4. pH-Sensitive Multistimuli-Responsive Hydrogels

Intelligent hydrogels that respond to multiple stimuli, particularly those sensitive to pH changes, have shown significant potential as new or auxiliary methods for tumor and wound treatments. However, they also face the challenge of adapting to complex and variable physiological conditions. Besides pH variations, many diseased tissues experience changes in ROS, enzymes, glucose levels, pressure, and temperature [52]. For instance, tumor cells can cause a decrease in local pH and an increase in temperature around the affected area [53]. Given these multifaceted changes, it becomes evident that single pH-responsive hydrogels are insufficient to adapt to diverse physiological responses, thereby limiting their therapeutic efficacy [54]. Consequently, developing pH-sensitive dual- or multistimuli-responsive hydrogels has become a focal point of current research efforts.

### 4.1. Dual-Responsive Hydrogels

Dual-responsive hydrogels are a type of hydrogel that can adapt to different physicochemical environments, thereby achieving more precise drug delivery. The response requirements for dual-responsive hydrogels are more stringent; structural changes occur within the hydrogel only when two conditions are simultaneously met, leading to swelling or degradation and thus releasing the drug [55]. The following sections will introduce pH-sensitive dual-responsive hydrogels in detail, which can be categorized into pH/temperature dual-responsive hydrogels, pH/redox dual-responsive hydrogels, pH/enzyme dual-responsive hydrogels, and pH/glucose dual-responsive hydrogels. The construction of these hydrogels offers several notable features: (1) targeted drug release for precise wound treatment, (2) enhanced mechanical properties with a higher Young’s modulus, and (3) self-healing capabilities.

#### 4.1.1. pH/Temperature Dual-Responsive Hydrogels

**Figure 7:**
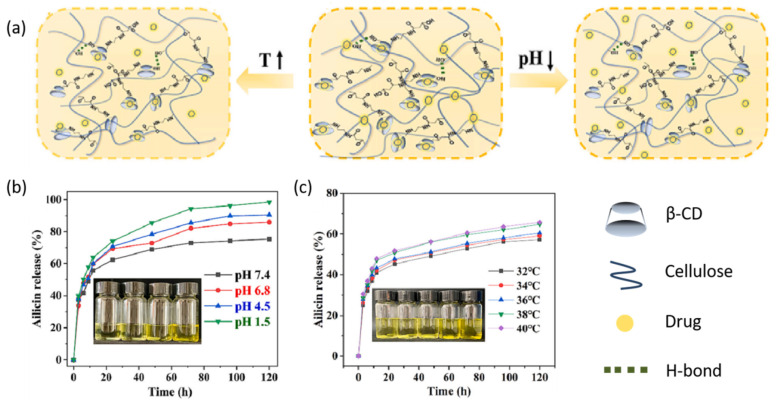
pH/temperature dual-responsive principle of crosslinked modified cyclodextrin–cellulose hydrogel. Drug release simulation of hydrogels in different environments (**a**); Drug release curves of BPCH-B at different pH (**b**) and temperatures (**c**) [58]. Copyright Zhang Y et al. Int. J. Biol. Macromol.

Infectious wounds caused by bacterial infections and hypoxic tumor tissues often exhibit acidic conditions, with local temperatures elevated above normal body temperature due to inflammation [56]. The design of pH/temperature dual-responsive hydrogels is particularly suited to addressing these conditions. Fathi M et al. [57] developed a temperature and pH dual-responsive hydrogel for breast cancer treatment. This hydrogel is formed by crosslinking poly(*N*-isopropylacrylamide) (PNIPAAm) and chitosan using glycerophosphate ions. In environments exceeding body temperature, the hydrophobic groups of PNIPAAm aggregate and expel water molecules, leading to the release of encapsulated drugs from the hydrogel. In vitro experiments demonstrated that this hydrogel releases the anticancer drug doxorubicin in the tumor cell environment (pH = 5.5, 40 °C), effectively killing tumor cells. Additionally, Zhang, Y. et al. [58] prepared a composite hydrogel loaded with berberine using bamboo cellulose and in situ crosslinked carboxylated β-cyclodextrin (BPCH-B) via a dissolution method. This dressing was shown to modulate drug release kinetics based on the pH and temperature conditions at the wound site (Figure 7a). Studies have indicated that at pH 7.4, BPCH-B released a significant amount of BBR (60%) within 70 h. As the pH decreases, the release of drug molecules from the gel accelerates; at pH 1.5, the gel releases up to 95% of the drug after 70 h (Figure 7b). This is due to the gradual breaking of entanglements between modified cyclodextrin-grafted cellulose chains under environmental diffusion, causing dissociation of the hydrogel network and making it easier for the drug to be released from the hydrogel. Additionally, it was observed that as the environmental temperature increased from 32 °C to 34 °C, 36 °C, and 38 °C, the drug release rate gradually increased, with a relatively faster drug release curve observed at 40 °C (Figure 7c). These results can be attributed to the contraction of the cellulose–cyclodextrin network chains as the temperature rises, facilitating easier release of the drug from within the hydrogel.

#### 4.1.2. pH/Redox Dual-Responsive Hydrogels

Metabolic reactions occur continuously within the human body [59,60], and in areas with high metabolic activity (such as tumors [61] and the intestines [62]), redox reactions occur more frequently than in other tissues [63], leading to changes in the local pH. Xu Y et al. [64] utilized periodate oxidation to crosslink hyaluronic acid (HA) via acylhydrazone and disulfide bonds, designing an injectable HA-CHO hydrogel. By varying the degree of oxidation, different hydrogels were created. It was found that increased oxidation led to higher mechanical strength and injection force. Additionally, the study confirmed that the hydrogel degraded faster under reducing conditions with dithiothreitol (DTT) and acidic conditions (pH = 2). This phenomenon may be primarily attributed to the progressive degradation of the hydrogel network through cleavage of the acid–labile acylhydrazone bonds under acidic conditions. The pH-dependent contraction of the hydrogel matrix creates a substantial concentration gradient between the internal network and the external medium, thereby enhancing the driving force for bovine serum albumin (BSA) release through accelerated diffusion kinetics. Furthermore, the introduction of the reducing agent dithiothreitol (DTT) induces complete hydrogel dissolution. This transition to a sol state results from the reductive cleavage of disulfide bonds, which serve as critical crosslinking nodes, leading to irreversible structural collapse of the three-dimensional polymeric network.

**Figure 8:**
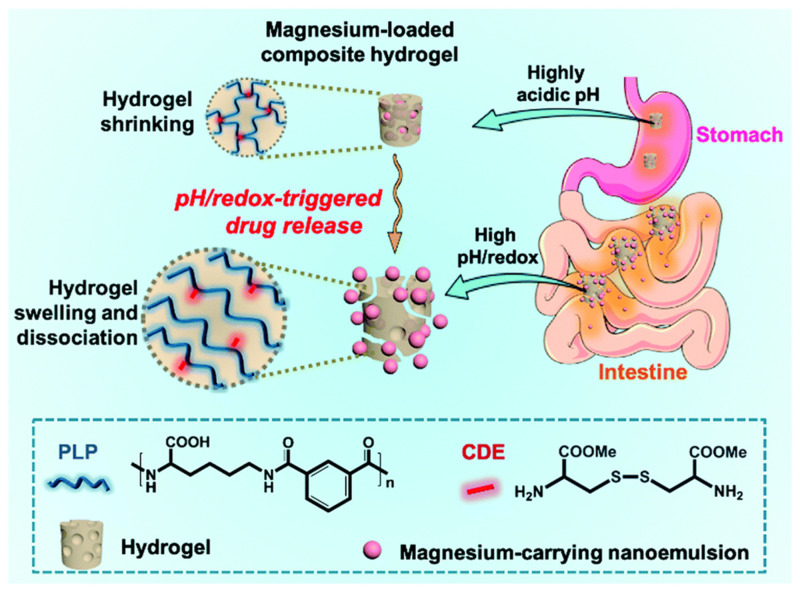
Schematic illustration of nanoemulsion-encapsulated hydrogels for effective oral drug delivery and triggered intestinal release of magnesium ions [65]. Copyright Huang Y et al. J. Mater. Chem. B.

Huang Y et al. [65] developed a pH/redox-responsive hydrogel (PLP) synthesized by crosslinking pH-responsive pseudopeptidepolylysineterephthalamide and redox-sensitive L-cystinedimethylesterdihydrochloride. They treated PLP hydrogels for 6 h using simulated gastric fluid (SGF, pH 1.2), simulated intestinal fluid (SIF, pH 6.8), and SIF (pH 6.8) + 1,4-dithiothreitol (a reducing agent). Due to the differing pH environments, the carboxyl groups of PLP deprotonated at a higher pH, causing hydrogel swelling. The presence of the reducing agent led to the cleavage of some internal disulfide bonds, further increasing hydrogel swelling and enabling targeted drug release. This demonstrated the dual pH/redox responsiveness of the hydrogel, as shown in Figure 8.

#### 4.1.3. pH/Enzyme Dual-Responsive Hydrogels

The pH level significantly influences enzyme activity. In the vicinity of wounds, infections by bacteria and viruses can occur. During their proliferation, these pathogens cause a decrease in the microenvironment’s pH [66], which in turn enhances enzyme activity [67]. Scientists have explored utilizing these enzymes to catalyze hydrogels for controlled drug release, aiming to achieve therapeutic effects.

**Figure 9:**
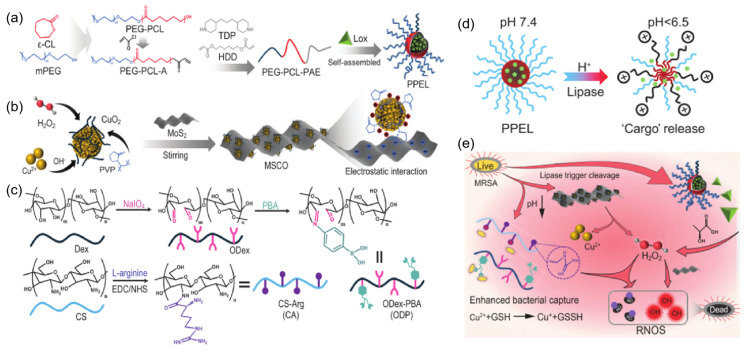
Schematic illustration of the preparation and application of pH/enzyme dual-responsive hydrogel. (**a**) Synthesis of Lox- loaded PPE micelle (PPEL). (**b**) Synthesis of MSCO nanozyme. (**c**) Synthesis of ODex-PBA (ODP) and CS-Arg (CA). (**d**) Mechanism of pH/lipase-responsive ‘cargo’ release of PPEL micelle. (**e**) The antibacterial mechanism of hydrogels in infected wounds [68]. Copyright Yang YT et al. National Science Review.

Guo Baolin et al. [68] designed and fabricated a series of intelligent self-activating, on-demand antibacterial hydrogels responsive to the bacterial-infected microenvironment. The researchers first synthesized pH/lipase-responsive polyethylene glycol-polycaprolactonepoly-β-aminoester (PEG-PCL-PAE, PPE) triblock copolymer micelles to encapsulate lactate oxidase (Lox) (Figure 9a). Subsequently, they developed pH-responsive hydrogen peroxide (H_2_O_2_)-self-supplying chemodynamic therapy donors, CuO_2_ nanoparticles, which were anchored onto the surface of MoS_2_ nanosheets via electrostatic interactions to form composite nanozymes (MSCO) (Figure 9b). Finally, they constructed a self-healing hydrogel through dynamic Schiff base bonds and phenylboronate ester linkages using L-arginine-modified chitosan (CA) and phenylboronic acid-functionalized oxidized dextran (ODP) (Figure 9c). By encapsulating MSCO nanozymes and PPE micelles loaded with Lox (PPEL), the hydrogel achieved self-recognition and self-treatment of infections. In the acidic microenvironment of bacterial infection, the hydrogel’s network structure collapses due to the cleavage of acid-labile Schiff base bonds and phenylboronate ester linkages, resulting in the release of PPEL micelles and MSCO nanozymes. In the presence of acidic metabolites and lipase, PPEL releases the encapsulated Lox (Figure 9b). Lox catalyzes the decomposition of lactate to generate H_2_O_2_, which synergizes with the H_2_O_2_ produced by MSCO nanozyme hydrolysis to promote the catalytic conversion of L-arginine (in CA) into nitric oxide (NO). Under H_2_O_2_-rich conditions, the peroxidase (POD)-like activity of MoS_2_ enhances the Fenton reaction catalyzed by Cu²⁺, generating reactive oxygen species (ROS) that, together with NO, effectively eliminate bacteria. Additionally, MSCO exhibits glutathione (GSH)-scavenging activity through the release of Cu²⁺, further compromising bacterial defenses against ROS (Figure 9e). Collectively, the engineered composite hydrogel demonstrates pH/enzyme-responsive, self-activating, and on-demand antibacterial capabilities.

#### 4.1.4. pH/Glucose Dual-Responsive Hydrogels

**Figure 10:**
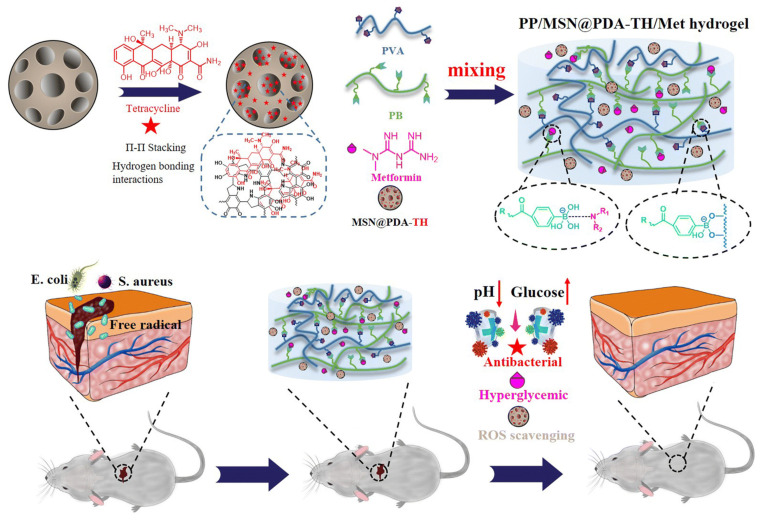
Preparation principle of polypropylene/MSN@PDA hydrogel and experimental process on mice [71]. Copyright Zhang W et al. J. Mater. Chem. B.

Diabetic complications can lead to foot ulcers [69]. To alleviate the suffering of diabetic patients with foot ulcers, Zhao Let al. [70] prepared a CSPBA/PVA/OHC-PEG-CHO hydrogel. Experimental findings revealed that under conditions of pH 7.4 and in the presence of glucose, the drug release from this hydrogel was significantly better compared to other individual conditions (pH 7.4 or pH 6.5). Zhang Wet al. [71] developed a hybrid diabetic wound dressing system integrating drug-loaded mesoporous silica, injectable polymer hydrogel, and hypoglycemic agent metformin (Met). The system was constructed by first mixing poly(acrylamide-co-dimethylaminopropylacrylamide-co-methacrylamidophenylboronic acid) (abbreviated as PB) with polyvinyl alcohol (PVA) to form an injectable pH/glucose dual-responsive hydrogel (designated as PP) through dynamic phenylboronate–diol bonds between PB and PVA. Meanwhile, polydopamine-modified mesoporous silica nanoparticles (MSN@PDA) were synthesized to load tetracycline hydrochloride (TH), yielding drug-carrying MSN@PDA-TH nanoparticles. The final hybrid hydrogel (abbreviated as PP/MSN@PDA-TH/Met) was obtained by blending PB, PVA, Met, and MSN@PDA-TH. Experimental results demonstrated that phenylboronate ester bond cleavage under acidic conditions (pH = 5.0) triggered partial hydrogel degradation and drug release. Additionally, competitive binding between phenylboronic acid groups and glucose 1,2-diol structures enhanced degradation under hyperglycemic conditions. Animal studies have confirmed the hydrogel’s efficacy in promoting skin ulcer healing in diabetic mice. The fabrication mechanism and experimental workflow are illustrated in Figure 10.

### 4.2. Triple-Responsive Hydrogels

The diversity and complexity of human lesions make it extremely challenging to achieve comprehensive and systematic treatment using a single type of gel delivery system. However, multi-responsive hydrogels, which can alter their volume, shape, mechanical, or chemical properties in response to environmental changes, effectively address this challenge by enabling diverse functionalities [72,73,74]. In 2007, the German researcher Bhattacharya S. [75] first proposed the preparation of polymer microgels with core–shell structures and pH/temperature responsiveness through batch copolymerization of *N*-vinylcaprolactam (VCL), acetoacetoxyethyl methacrylate (AAEM), and vinylimidazole (VIm). Using these microgels as templates, magnetite nanoparticles were subsequently generated in situ to create hybrid microgels. This approach developed monodisperse, colloidally stable microgel particles exhibiting triple-responsive properties to temperature, pH, and magnetic fields. Such microgel particles hold great promise as ideal materials combining tumor thermotherapy with controlled drug release systems.

**Figure 11:**
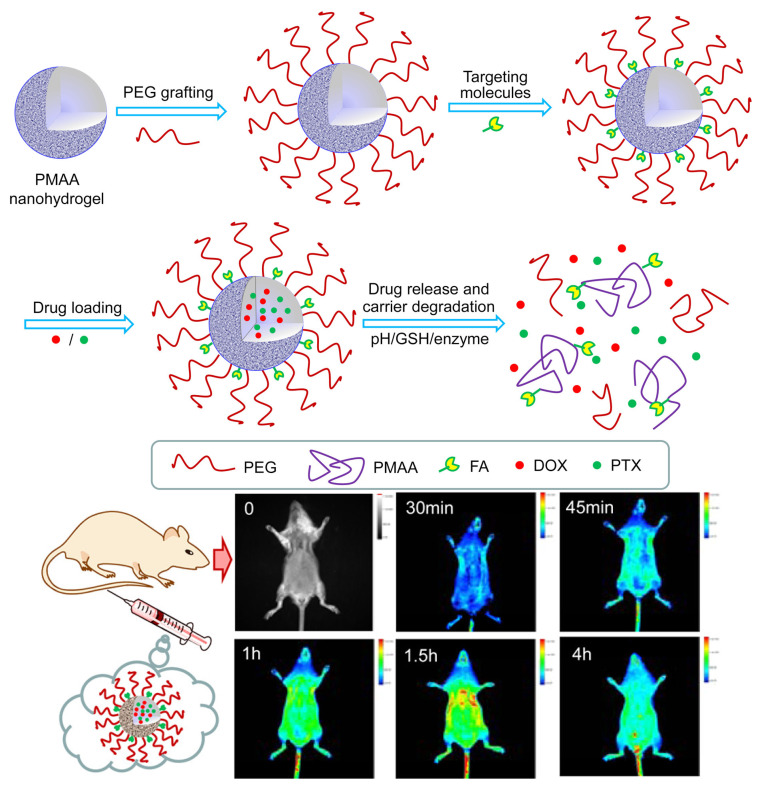
Preparation of FA and PEG-modified PMAA nanohydrogels, drug loading and release, and biocompatibility assessment [76]. Copyright Jin S et al. ACS Appl. Mater. Interfaces.

The development of nanogel materials in smart drug delivery for cancer treatment has advanced rapidly.Jin S et al. [76] synthesized a novel protease/redox/pH-responsive biodegradable nanohydrogel using methyl methacrylate (MAA) as the monomer and N,N-bis(acryloyl) cystamine (BACy) as the crosslinker. As shown in Figure 11, polyethylene glycol (PEG) and folic acid (FA) were covalently grafted onto the surface of the nanohydrogel to enhance its prolonged in vivo circulation time and active targeting ability towards tumor cells and tissues. This nanohydrogel can respond to glutathione (GSH) through reduction-sensitive disulfide bonds and can also respond to proteases by disrupting the amide bonds in the crosslinked network. The nanohydrogel was used to simultaneously load the amphiphilic drug doxorubicin (DOX) and hydrophobic drug paclitaxel (PTX), exhibiting high drug loading efficiency. Cumulative release curves demonstrated that weak acidic (pH 5.0) and reducing (GSH) environments significantly accelerated the drug release from the drug-loaded nanohydrogel, reducing drug leakage before reaching the tumor site. In addition, in vivo fluorescence imaging studies were conducted to evaluate the biocompatibility of the nanohydrogel.

## 5. Applications of pH-Responsive Hydrogels

pH-responsive hydrogels not only are excellent carriers for cells, antibiotics, growth factors, and biomacromolecules but also exhibit morphological changes, swelling behavior, degradation, or drug delivery efficiency in response to specific environmental triggers. Consequently, they have been widely applied in various medical fields such as controlled drug release [77], wound tissue repair [78], tumor-targeted therapy [79], and biosensing [80,81]. In this section, we will discuss the applications of pH-responsive hydrogels in different medical scenarios.

### 5.1. Innovations in Drug Delivery Systems

The core advantage of pH-responsive hydrogels in drug delivery lies in their ability to achieve targeted and on-demand release, thereby reducing systemic toxicity and enhancing therapeutic efficacy [82]. Additionally, for hydrogels at the micron and nanometer scales, their high water content can serve as a stealth structure, which helps in evading host immune responses, thereby increasing the in vivo circulation time [83,84,85]. In recent years, researchers have utilized various natural or synthetic polymer materials to fabricate pH-responsive hydrogels. These hydrogels exhibit swelling or degradation behavior in different pH environments, allowing for the release of various drugs (as shown in Table 1).

The most common application of pH-responsive hydrogels is in oral drug delivery. The pH range in the oral cavity is 6.7 to 7.3 [93], while the gastrointestinal tract exhibits a significant pH gradient (stomach pH 1.5–3.5, intestine pH 6.5–7.4), providing a natural setting for pH-responsive hydrogels. For instance, Phan V.H.G. et al. [94] developed chitosan/alginate composite hydrogels that remain stable in the acidic gastric environment and dissolve upon entering the intestine due to the increased pH, enabling the delivery of protein-based drugs such as insulin without degradation by gastric acid. Similarly, Xu Q et al. [95] prepared poly(methacrylic acid) (PMAA)-based hydrogels that exhibit pH-dependent swelling behavior through carboxyl group protonation-deprotonation, which can be used for colon-targeted delivery of anti-inflammatory drugs like 5-aminosalicylic acid. In targeting the tumor microenvironment, where enhanced glycolytic metabolism results in a slightly acidic condition (pH 6.5–6.9), pH-responsive hydrogels can achieve precise chemotherapy. Zeoliticimidazolate framework−8 (ZIF−8), a novel metal-organic framework (MOF) material with adjustable pore size, high porosity, good biocompatibility, and stability, has been utilized in this context. By loading doxorubicin (DOX) into ZIF-8 nanoparticles and embedding them within a pH-responsive hydrogel network, MOF-composite hydrogels can be fabricated. Upon entering the tumor microenvironment, acidic conditions trigger the decomposition of ZIF−8 and release of the drug, significantly increasing the drug concentration at the tumor site [96].

### 5.2. Tissue Engineering and Regenerative Medicine

pH-responsive hydrogels intended for use in the human body not only require biochemical properties such as chemical bonding with tissues and reliable biocompatibility but also need to possess certain three-dimensional structures and biophysical characteristics (shape, mechanical strength, and permeability). This ensures that they can not only serve as scaffold materials for tissues and organs but also dynamically respond to microenvironmental changes to promote cell proliferation, differentiation, and tissue repair [97,98,99,100]. 3D-printed hydrogels represent an emerging field in bioengineering. Compared to hydrogels prepared using traditional methods, 3D bioprinting allows for the direct printing of cells within the hydrogel matrix, thus enhancing its extensibility. This promotes cell adhesion and infiltration into the printed structure, thereby restoring function in injured tissues [101,102]. Consequently, 3D-printed hydrogels have found broad applications in repairing bone and joint tissues, combating infections, treating tumors [103,104,105], and constructing nerve guidance conduits [70].

Currently, mimicking the complex structures of natural blood vessels and constructing vascular networks within tissue engineering scaffolds remains challenging. Researchers have addressed this by integrating stimuli-responsive hydrogels with 3D printing technology to fabricate independent multi-branched vessels and complex vascular networks within heterogeneous porous scaffolds. By leveraging the sol–gel transition of temperature-responsive gelatin and the dual physical crosslinking networks of pH-responsive chitosan (i.e., the electrostatic network between protonated chitosan and sulfate ions and the crystalline network of neutral chitosan), physiologically stable gelatin/chitosan hydrogel tubes can be constructed. Research data indicate that these artificial blood vessels (gelatin/chitosan hydrogel tubes) exhibit excellent physiological stability, mechanical strength, semi-permeability, and cell compatibility and a low in vivo inflammatory response [106].

### 5.3. Diagnostics and Biosensing

The pH value is a critical determinant controlling the metabolic rate during the wound-healing process and is an important parameter for therapeutic intervention in wound care [107,108]. Traditional pH-responsive hydrogels used in diagnostics primarily rely on swelling-induced changes in the refractive index of the hydrogel [109] or the incorporation of chemical indicators to provide qualitative analysis of the wound-healing process. For instance, Tsegay F et al. [110] prepared a hydrogel resin (HEMA/PEGDA/TPO/AA-PR) using an alcohol-based hybrid method, incorporating phenol red (a pH-responsive sensor) into wound dressings. The color change reflects the pH variations in the wound, indirectly indicating the extent of wound healing.With technological advancements, more sophisticated methods involve using fluorescence for the quantitative monitoring of wound infection or healing. Xiao J.Y. et al. [111] developed a microneedle patch integrated with pH-responsive hydrogels to achieve real-time detection of pH changes in wounds (pH elevation at infected sites). By integrating pH-sensitive fluorescent indicators into the PVA gel base of the microneedles, this patch exhibits excellent antibacterial properties, reliable fluorescence sensing capabilities, and high predictive accuracy for quantitative analysis of wound fluid pH. This combination of machine learning algorithms with microneedle sensing patches provides a flexible and reliable strategy for advanced wound management and point-of-care diagnostics.

In addition to wound monitoring, pH-responsive intelligent hydrogels can be utilized for detecting and analyzing bodily fluid components. Through receptor immobilization and metal ion treatment functionalization, photonic cholesteric liquid crystal solid (CLCsolid) droplets can be interwoven with a polyacrylic acid (PAA) network featuring an interpenetrating polymer network (IPN) structure, forming photonic IPN-CLCsolid-PAA droplets. These photonic IPN-CLCsolid-PAA droplets serve as individual sensors within patterned array PAA films. Upon responding to pH variations, these droplets exhibit observable changes in the reflected central wavelength color. By immobilizing urease and phenylboronic acid on the PAA network, 10-micrometer-sized photonic IPN-CLCsolid-PAA droplets can be implemented in optical photonic biosensors. The compartmentalized photonic IPN-CLCsolid-PAA droplets in this patterned array film enable multiplex detection of pH, divalent metal ions, urea, and glucose, thereby facilitating applications in sports health monitoring [112].

## 6. Advanced Materials and Technological Innovation

### 6.1. Multifunctional Integrated Hydrogels

It is well known that diabetic foot ulcers (DFUs) are notoriously difficult to heal due to severe infections, excessive inflammation, and challenges in angiogenesis [113,114]. Combining bio-organic polymers with inorganic nanoparticles to form novel composite gels can synergistically leverage both chemical reactions and physical treatments, offering a promising approach for addressing complex chronic diseases like DFUs.

For instance, researchers have developed a pH/glucose dual-responsive hydrogel dressing (HPC) by bifunctionalizing hyaluronic acid (HA) and oxidized chondroitin sulfate (OCS) through aminophenylboronic acid and adipicdihydrazide, forming Schiff base and phenylboronate ester dual dynamic crosslinks. Subsequently, polydopamine-reduced graphene oxide-glycine-functionalized fullerene (GPC), which exhibits photothermal/photodynamic synergistic antibacterial properties, along with pirfenidone (PFD), a drug that promotes angiogenesis and inhibits inflammation, was incorporated into the HPC hydrogel.Based on the pH/glucose responsiveness of the Schiff base/phenylboronate ester, the HPC/GPC/PFD (HPCG/PFD) hydrogel can accelerate the release of PFD, thereby alleviating excessive inflammation and promoting angiogenesis in DFUs. Additionally, dual dynamic crosslinking endows the hydrogel with excellent on-demand removal and self-healing properties, while GPC enhances the hydrogel’s tissue adhesion, antioxidant capacity, and electrical conductivity. In a rat DFU model, this hydrogel has been shown to promote wound healing by reducing infection and inflammation, accelerating wound closure and enhancing epidermal regeneration, collagen deposition, and angiogenesis, thus facilitating diabetic wound recovery [115].

### 6.2. Biomimetic Design

Cell-membrane biomimetic-coated nanoparticles (CNPs) represent a promising nanocarrier platform with unique biological properties derived from biomimetic design [116,117]. Featuring a core–shell structure, the gel-based core is enveloped by a cell membrane camouflage coating. This architecture not only enables immune evasion but also achieves homologous targeting to lesion sites through adhesion molecule mediation [118,119].

Shang L. et al. [120] innovatively developed a cancer cell membrane (CCM)-coated pH-responsive nanogel (NG@M). The nanogel core comprises poly(2-diethylaminoethyl acrylate) (PDEA), hydroxypropyl-β-cyclodextrin (HP-β-CD), and Pluronic F127 copolymer, encapsulated by a cancer cell membrane coating. In the tumor microenvironment (pH 6.5), protonation of tertiary amine groups in PDEA triggers core expansion and disintegration. This volumetric expansion generates increased osmotic pressure within the membrane coating, leading to structural disruption and site-specific drug release. Experimental results demonstrate 85.9% and 84.7% release rates for paclitaxel (PTX) and interleukin−2 (IL−2) within 12 h, respectively. The released PTX exerts cytotoxic effects via passive diffusion into tumor cells, while IL−2 enhances T-cell proliferation and activation to amplify immune responses. Their synergistic action significantly improves antitumor efficacy, offering an innovative delivery strategy for combined cancer immunotherapy.

### 6.3. Microfluidic Technology

Microfluidic technology is an innovative interdisciplinary field that has evolved from microelectronics, micromechanics, bioengineering, and nanotechnology. It focuses on controlling, manipulating, and analyzing complex fluids at microscopic scales. By constructing microfluidic channel systems, this technology enables intricate fluid manipulations, making it increasingly popular in the synthesis of nanomaterials and biomedical research [121]. Microfluidic channels are typically at the micrometer scale, allowing precise control of fluid flow rates, mixing ratios, and reaction times. This enables high-level control over the size, morphology, porosity, and mechanical properties of hydrogels. Moreover, through multi-channel design or gradient mixing, hydrogels with core–shell structures, porous networks, and heterogeneous compositions, or even those integrated with cells or drugs, can be prepared, enabling functional customization (such as targeted sustained release) [122].

Chitosan and alginate hydrogels, due to their high biocompatibility [123], are ideal for developing nanodrug microcapsules. As a result, they hold significant appeal in drug delivery systems. Liu R. et al. [124] developed a PDMS-based droplet microfluidic reactor for synthesizing hydrogel microcapsules. The synthesized microcapsules include Au@CoFeB-Rg3 as a model for nanodrugs and a hydrogel model composed of sodium alginate and PEG-g-chitosan mixture crosslinked by genipin. By simulating the pH and temperature conditions of the gastrointestinal tract during drug transport, as well as the pH and temperature of the target pathological cell microenvironment, the release kinetics of the nanodrug from the hydrogel were evaluated. The results showed that the nanodrug-loaded hydrogel could rapidly degrade at pH 5.5 and remain relatively stable at 37 °C and pH 7.4, proving that the designed nanodrug hydrogel microcapsule system is suitable for oral administration.

## 7. Conclusions and Prospects

In recent years, pH-responsive hydrogels have been widely applied in the field of biomedicine, exhibiting many excellent properties. This paper mainly discusses the principles, types, advantages, and applications of pH-responsive hydrogels, as well as the development trend from single-response to dual-response and multi-response pH-sensitive gels. It also summarizes the methods and processes for preparing pH hydrogels. Through the discussion of the above content, the significant role of pH-responsive hydrogels in the biomedical field is highlighted. Despite the many advantages of pH-responsive hydrogels, there are still some challenges that need further investigation, such as the relatively single stimulus source for shape memory, long self-repair time, and so on. Regarding biocompatibility, the residuals of synthetic materials (such as acrylate monomers) may trigger inflammatory responses, requiring further optimization of the polymerization process and purification methods. Additionally, the complex ionic strength and protein adsorption in the physiological environment may interfere with the pH-responsive behavior of hydrogels. Whether the stability of these hydrogels in the physiological environment can be enhanced by introducing anti-fouling coatings (such as PEG) or nanocomposite structures remains an area for further research. Moreover, many pH-responsive hydrogels remain in the experimental stage and require continuous testing before they can be brought to market. Therefore, their path to commercialization is long and complex. This process necessitates further integration of technology and continuous innovation from both the pharmaceutical and engineering sectors. It also requires a standardized clinical trial process and the coordination of supporting commercial-scale operations to work together effectively.

In the future, with the advancement of material science and advanced manufacturing processes (such as microfluidics and 3D printing), along with the integration of intelligent systems like AI-driven design (using machine learning to predict the relationship between material structure and performance), pH-responsive hydrogels as smart biomaterials are gradually transitioning from the laboratory to clinical applications. Their application scenarios are expanding into precision medicine, regenerative medicine, and personalized diagnostics, thereby providing more efficient and safer therapeutic solutions for human healthcare.

## Figures and Tables

**Table 1:** Summary of pH-responsive hydrogels in drug delivery applications.

Composition	Application	Response Conditions	Mechanism of pH Response	Ref.
GelMA, HA-CHO, SEBS	Release of antibiotic: GS and LZM	pH = 5.0	The cleaved Schiff base bonds and the electrostatic interactions	[86]
SiNPs, alginates	Release of antibiotic: CHX	pH > 8.0	Microporous SiNPs is a simple diffusion of the loaded compounds or a degradation of the silica matrix	[87]
GelMA, PeMA	Release of natural antibacterial substance: curcumin	pH = 7.4	Carboxylic acid groups on both PeMA and GelMA and pH-dependent solubility of curcumin	[88]
CS, OSU	Release of natural antibacterial substance: MAG	pH = 6.2	pH-responsive swelling behavior	[89]
NIPAAM, AAM, DMPA, methylene-N,N-bis(acrylamide)	Antibiotics (Kana) are used within the hydrogel to kill bacteria through Bacterial trap behavior and Fenton reaction	pH = 5.5	The responsive released Cu^2+^ in the weak acidic environment can catalyze the decomposition of self-supplied H_2_O_2_ into •OH.	[90]
ARX, CS, rGO, TEOS	Delivery of antibiotic: silver-sulphadiazine	pH = 7.4	The protonation of the alcoholic and carboxylic acid functional groups of ARX and CS	[91]
CS, PNIAAm-co-IA, GP	Release antitumor drugs: DOX	pH = 5.5, 40 °C	Result in preventing the proper interaction of functional groups of CS in the acidic pH	[57]
Bamboo parenchymal cellulose, carboxylated-β-cyclodextrin	Release antimicrobials: BBR	pH = 7.6, 40 °C	The entanglements between the modified cyclodextrin-grafted cellulose chains are gradually broken	[58]
Self-assembled peptide, NaCl	Release antitumor drugs: PTX	pH = 5.8, GSH	The three-dimensional network is damaged under the conditions of slight acidity	[92]
PLP, CDE	Deliver Mg^2+^	pH = 6.8, DTT	Protonation/deprotonation of carboxyl (-COOH) groups and cleavage of disulfide bonds	[65]
PPE, CS, ODP, MSCO	Release Lox to break down lactic acid produced by bacteria, achieving an antibacterial effect.	pH=5.5, lipase	The cleaved Schiff base bonds in the acidic pH	[68]
Met, PVA, mesoporous silica nanoparticles (MSN@PDA), PB	Release hypoglycemic drugs: Met Release antimicrobials: TH	pH = 5.0, glucose	The phenyl borate group is unstable at acidic pH	[71]

## Data Availability

No new data were created or analyzed in this study.

## Abbreviations

AAEM	Acetoacetoxyethyl methacrylate
AAM	Acrylamide
ADR	Anthracycline
ARX	Arabinoxylan
BACy	N,N-bis(acryloyl) cystamine
BBR	Berberine
BC	Bacterial cellulose
BPCH-B	Bamboo cellulose and in situ crosslinked carboxylated β-cyclodextrin
BSA	Bovine serum albumin
CA	L-arginine-modified chitosan
CCM	Cancer cell membrane
CDE	L-cystine dimethyl ester dihydrochloride
CHX	Chlorhexidine;methacrylated pectin
CLC	Cholesteric liquid crystal
CLCsolid	Cholesteric liquid crystal solid
CNPs	Cell membrane-coated nanoparticles
CS	Chitosan
DFUs	Diabetic foot ulcers
DMPA	N-[3-(dimethylamino)propyl]methacrylamide
DOX	Doxorubicin
DTT	Dithiothreitol
FA	Folic acid
GelMA	Gelatin methacryloyl
GO	Graphene oxide
GP	Glycerophosphate
GS	Gentamicin sulfate
GSH	Glutathione
HA	Hyaluronic acid
HA-CHO	Hyaluronic acid aldehyde
HP-β-CD	hydroxypropyl-β-cyclodextrin
IL	Interleukin
IPN	Interpenetrating polymer network
Kana	Kanamycin
Lox	lactate oxidase
LZM	Lysozyme
MAG	Magnolol
Met	Metformin
MMA	Methyl methacrylate
MOF	Metal–organic framework
MSCO	composite nanozyme
MSN@PDA	Polydopamine-modified mesoporous silica nanoparticles
NIPAAM	N-isopropylacrylamide
OCS	Oxidized chondroitin sulfate
ODP	phenylboronic acid modified oxidized dextran
OSU	Oxidized sucrose crosslinker
PAA	Polyacrylic acid
PB	Poly(acrylamide-co-dimethylaminopropylacrylamide-co-methacrylamidophenylboronic acid)
PDEA	Poly(2-diethylaminoethyl acrylate)
PED	Pirfenidone
PEG	Polyethylene glycol
PeMA	Methacrylated pectin
PLP	Poly(l-lysine isophthalamide)
PMAA	Poly(methacrylic acid)
PNIAAm	Poly(N-isopropylacrylamide)
PPE	polyethylene glycol-polycaprolactone-poly-β-aminoester triblock copolymer micelles
PPEL	PPE micelles loaded with Lox
PTX	Paclitaxel
PVA	Polyvinyl alcohol
ROS	Reactive oxygen species
SEBS	Poly(styrene-b-(ethylene-co-butylene)-b-styrene)
SGF	Simulated gastric fluid
SIF	Simulated intestinal fluid
SiNPs	Silica nanoparticles
TEOS	Tetraethyl orthosilicate
TH	Tetracycline hydrochloride
VCL	N-vinylcaprolactam
VIm	Vinylimidazole
ZIF−8	Zeolitic imidazolate framework−8
